# Enhanced Association of Novel Cardiovascular Biomarkers Fetuin-A and Catestatin with Serological and Inflammatory Markers in Rheumatoid Arthritis Patients

**DOI:** 10.3390/ijms25189910

**Published:** 2024-09-13

**Authors:** Anna Pàmies, Dídac Llop, Daiana Ibarretxe, Roser Rosales, Josefa Girona, Lluís Masana, Joan-Carles Vallvé, Silvia Paredes

**Affiliations:** 1 Secció de Reumatologia, Hospital Verge de la Cinta, 43500 Tortosa, Spain; pamies.anna@gmail.com; 2 Unitat de Recerca en Lípids i Arteriosclerosi, Universitat Rovira i Virgili, 43201 Reus, Spain; didac.llop@urv.cat (D.L.); daiana.ibarretxe@urv.cat (D.I.); roser.rosales@ciberdem.org (R.R.); josefa.girona@urv.cat (J.G.); luis.masana@urv.cat (L.M.); silvia.paredes@salutsantjoan.cat (S.P.); 3 Institut Investigació Sanitaria Pere Virgili, 43204 Reus, Spain; 4 Centro de Investigación Biomédica en Red de Diabetes y Enfermedades Metabólicas Asociadas (CIBERDEM), Instituto de Salud Carlos III (ISCIII), 28029 Madrid, Spain; 5 Unitat Medicina Vascular i Metabolisme, Hospital Universitari Sant Joan de Reus, 43204 Reus, Spain; 6 Secció de Reumatologia, Hospital Universitari Sant Joan de Reus, 43204 Reus, Spain

**Keywords:** rheumatoid arthritis, biomarkers, atherosclerosis, cardiovascular disease

## Abstract

Rheumatoid arthritis (RA) is a chronic autoimmune disease associated with increased cardiovascular disease (CVD) risk and mortality. This work aimed to evaluate the serum levels of the novel CV biomarkers fetuin-A (fet-A), Dickkopf-1 (DKK-1), galectin-3 (Gal-3), interleukin-32 (IL-32), and catestatin (CST) in RA patients and their associations with RA parameters and CVD markers. A cohort of 199 RA patients was assessed for traditional CVD risk factors, RA disease activity, and biomarker levels. Carotid ultrasound was used to measure carotid intima-media thickness (cIMT) and carotid plaque presence (cPP). Multivariate analyses examined correlations between biomarkers and RA parameters, serological markers, and CVD markers. Adjusted models showed that elevated CST expression levels were associated with rheumatoid factor (RF) and anti-citrullinated protein antibody (ACPA) positivity (OR = 2.45, *p* = 0.0001 and OR = 1.48, *p* = 0.04, respectively) in the overall cohort and for RF in men and women, respectively. In addition, fet-A concentration was inversely associated with the erythrocyte sedimentation rate (ESR) in the overall cohort (β = −0.15, *p* = 0.038) and in women (β = −0.25, *p* = 0.004). Fet-A levels were also negatively correlated with disease activity (DAS28-ESR) scores (β = −0.29, *p* = 0.01) and fibrinogen concentration (β = −0.22, *p* = 0.01) in women. No adjusted associations were observed for Gal-3, DKK-1 or IL32 concentration. The study revealed no significant associations between the biomarkers and cIMT or cPP. The measurement of CST and fet-A levels could enhance RA patient management and prognosis. However, the utility of biomarkers for evaluating CV risk via traditional surrogate markers is limited, highlighting the need for continued investigations into their roles in RA.

## 1. Introduction

Rheumatoid arthritis (RA) is a persistent, systemic autoimmune disorder primarily targeting the synovial joints. Its prevalence in the general population is estimated to be around 0.5–1%, with women being more frequently affected than men [1]. Without adequate treatment, the disease evolves along a progressive course of joint destruction, deterioration of physical function, and deterioration of quality of life for most patients [2]. Furthermore, individuals with RA experience a shorter life expectancy and higher mortality rates compared to the general population. The inflammatory activity of the disease also leads to extra-articular manifestations, including pulmonary, ocular, hematologic, and cardiovascular (CV) disease, with the latter being the leading cause of death in RA patients [3,4,5].

The diagnosis of RA is based on several parameters, including clinical symptoms, imaging features, and the detection of analytic inflammatory variables and serological markers such as rheumatoid factor (RF) and anti-citrullinated peptide/protein antibodies (ACPAs) [6]. RF seropositivity in patients with RA is associated with increased mortality from all causes, and the presence of ACPAs is associated with increased CV-related mortality [7].

**Figure 1:**
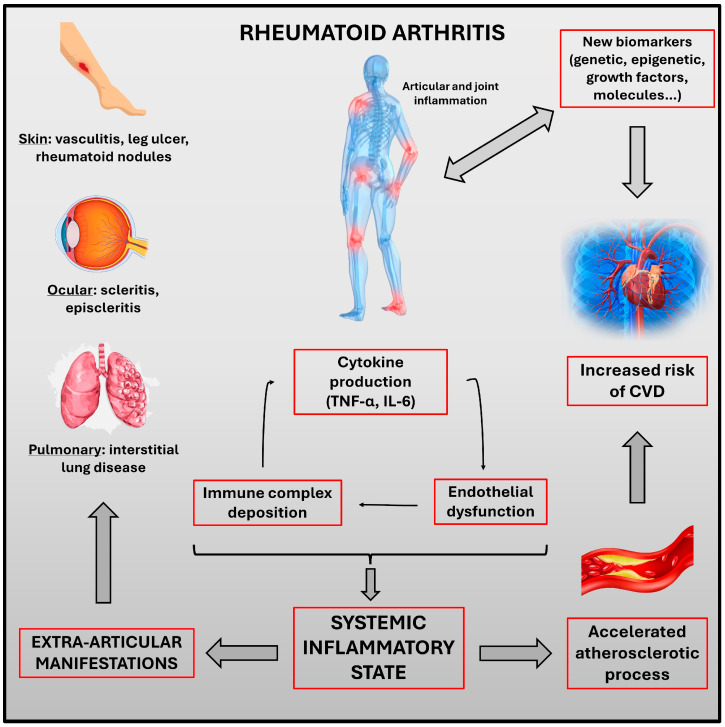
Rheumatoid arthritis: pathophysiological links between systemic inflammation and extra-articular manifestations.

Cardiovascular disease (CVD) represents the leading cause of both morbidity and mortality among patients with RA [8]. The risk of developing CVD in RA patients is estimated to be about 50% greater compared to the general population [8,9,10]. Both traditional CV risk factors (age, sex, smoking status, type 2 diabetes mellitus (T2D), and hypertension) and systemic inflammation contribute to the increase in CVD incidence in this population [11]. The underlying reasons for the heightened incidence of CVD in RA patients remain not fully understood. However, the inflammatory processes occurring in synovial membranes and atherosclerotic plaques share similar features [12]. Furthermore, in RA patients, there is an increase in the synthesis of proinflammatory cytokines, which facilitates endothelial dysfunction, a key initial step in the pathophysiology of atherosclerosis [13] (Figure 1).

Carotid intima-media thickness (cIMT), as measured by carotid ultrasound, is a well-established surrogate marker for arteriosclerosis and has been shown to predict CV events both in the general population [14,15] and in RA patients [16]. In RA patients, a cIMT exceeding 0.91 mm has been linked to an increased risk of CVD [17]. Additionally, the presence of carotid artery plaques (cPP) serves as a predictor of CV events in the general population [18]. In longitudinal studies, cPP in RA patients has been correlated with the occurrence of cardiovascular events and mortality [19].

Early diagnosis of CVD in patients with RA is essential for preventing its consequences and for implementing possible therapeutic strategies to reduce future CV events. Thus, identifying new biomarkers that can predict future CVD in patients with RA is crucial. New biomarkers could be useful for predicting CV risk and disease progression and could become therapeutic targets [20].

New CVD biomarkers (fetuin A (fet-A), Dickkopf Wnt signaling pathway inhibitor 1 (DKK-1), galectin 3 (Gal-3), interleukin-32 (IL-32), and catestatin (CST)) have recently been identified. Fet-A is a glycoprotein known for its anti-inflammatory and anticalcification properties [21,22]. Elevated serum levels of fet-A have been linked to reduced all-cause mortality in individuals with coronary heart disease [23]. Conversely, low levels of fet-A, coupled with increased C-reactive protein (CRP) levels, have been associated with an increased risk of CV-related mortality in patients experiencing acute coronary syndrome [24]. CST is a protein derived from chromogranin A (CgA). Elevated plasma levels of CgA have been observed in patients with CVD and hypertension, while low CST levels are linked to essential hypertension. CST mitigates hypertension by promoting histamine release and nitric oxide production and inhibiting catecholamine secretion [25]. Reduced conversion of CgA to CST is associated with increased mortality in patients with acute heart failure. Likely as a compensatory mechanism to inhibit catecholamine release, elevated CST levels have been found in patients with acute myocardial infarction [26], heart failure [27], and atrial fibrillation [28]. The prognostic value of CST levels in CVD patients remains controversial: some studies have linked low CST levels to poorer outcomes [29], while others have associated higher CST levels with adverse outcomes [30,31]. DKK-1 is a known inhibitor of the Wnt pathway and plays a critical role in endothelial dysfunction and atherosclerosis [32,33]. Elevated plasma levels of DKK-1 have been observed in patients suffering from stroke [34,35] and acute myocardial infarction [36], and these increased levels are correlated with poorer outcomes in these individuals [34,37,38]. Gal-3 is a β-galactoside-binding lectin that acts as a marker of fibrosis and plays a significant role in the immune response and inflammation [39]. Increased levels of Gal-3 have been associated with poor outcomes in patients with heart failure or stroke [40] and is considered a potential mediator of atherosclerosis [41]. Finally, IL-32 is a cytokine associated with increased CVD risk. Increased IL-32 levels in plasma and coronary artery lesions have been observed in ischaemic heart disease patients [42]. Moreover, high IL-32 levels predict worse outcomes in heart failure patients following acute myocardial infarction [43].

In the context of RA, recent data indicate that DKK-1 levels are associated with myocardial perfusion defects, suggesting a link between DKK-1 levels and CVD risk in RA patients [44]. Similarly, elevated Gal-3 levels have been associated with decreased cardiac output, increased systemic vascular resistance [45], and aortic inflammation, but not coronary calcification, in RA patients [46].

However, there are currently no data on the potential roles of fet-A, CST, or IL-32 as biomarkers for assessing cardiovascular risk in patients with RA.

In the present study, we aimed to evaluate the serum levels of these biomarkers (fet-A, DKK-1, Gal-3, IL-32, and CST) in RA patients and to assess their associations with RA parameters and with surrogate markers of CVD.

## 2. Results

### 2.1. General Characteristics of the RA Cohort

Table 1 presents the general characteristics of the RA cohort included in the study (n = 199), both overall and stratified by sex. The mean age of the cohort was 57.8 (12.4) years, with a median disease duration of 8 years (range: 3–13 years), and 66% of the participants were female. Of the total, 56% of the patients had high disease activity, while 44% had low disease activity. There were significantly more women with high disease activity than men. A total of 74.37% of the patients were positive for RF, and 84.42% were ACPA-positive. Among the patients, 75% received conventional synthetic disease-modifying antirheumatic drugs (csDMARDs), 57.28% received nonsteroidal anti-inflammatory drugs (NSAIDs), 51% received glucocorticoids, and 21.6% received biological disease-modifying antirheumatic drugs (bDMARDs). Additionally, 24.62% of patients were prescribed renin–angiotensin–aldosterone system (RAAS) inhibitors, 26.13% received other hypotensive treatments, 16.08% were on statins, and 0.15% were treated with other lipid-lowering drugs. No differences were observed between men and women in the levels of the studied biomarkers. Men in the RA cohort presented increased waist circumference, SBP, DBP, and cIMT and decreased HDL-C compared to women. Finally, the incidence of hypertension was greater in men than women, and DAS28-ESR values were greater in women than men.

Univariate analysis of the entire cohort revealed no significant correlation between most of the selected biomarkers and age, with only a modest correlation observed between age and fet-A (ρ = −0.14, *p* = 0.048) (Supplementary Table S1). Moreover, RF-positive patients exhibited elevated CST levels compared to RF-negative patients (*p* < 0.001). Furthermore, individuals with type 2 diabetes (T2D) displayed increased concentrations of Gal-3 (*p* = 0.02) compared to those without T2D, and those with high disease activity (DAS28-ESR ≥ 3.2) demonstrated higher levels of Gal-3 (*p* = 0.04) than patients with low disease activity (DAS28-ESR < 3.2). Conversely, fet-A concentration exhibited a negative correlation with CRP levels (ρ = −0.14, *p* = 0.04) and ESR (ρ = −0.15, *p* = 0.03) but showed a positive correlation with LDL-C levels (ρ = 0.17, *p* = 0.01). Finally, Gal-3 levels exhibited a positive correlation with HDL-C (ρ = 0.17, *p* = 0.01) (Supplementary Figure S1). After sex-stratified analyses were conducted, the following results were observed for male patients. Specifically, those with cPP and those with erosions displayed reduced levels of Gal-3 (*p* = 0.04 and *p* = 0.03, respectively). Furthermore, male patients with high disease activity exhibited elevated Gal-3 levels (*p* = 0.009). As for DKK1, increased levels were observed in RF-positive patients (*p* = 0.005).

Male patients with cPP had decreased levels of DKK-1 (*p* = 0.03), while patients with high disease activity had increased levels of DKK-1 (*p* = 0.02). RF-positive patients and those with cPP showed increased levels of CST (*p* = 0.02 and *p* = 0.04, respectively). Finally, positive correlations were identified between fet-A concentration and LDL-C levels (ρ = 0.34, *p* = 0.004), between CST concentration and LDL-C levels (ρ = 0.32, *p* = 0.009), between IL-32 concentration and LDL-C levels (ρ = 0.32, *p* = 0.009), and between DKK1 concentration and LDL-C levels (ρ = 0.27, *p* = 0.03) (Supplementary Figure S2). Among female patients, women with T2D displayed increased levels of CST (*p* = 0.04). Furthermore, RF-positive women also exhibited elevated levels of CST (*p* = 0.005). Negative correlations were noted between fet-A concentration and ESR (ρ = −0.25, *p* = 0.004), DAS28-ESR (ρ = −0.19, *p* = 0.03), and fibrinogen level (ρ = −0.24, *p* = 0.004) (Supplementary Figure S3).

Among the analysed biomarkers, IL-32 levels were positively correlated with CST concentration (ρ = 0.20, *p* = 0.003) and Gal-3 levels (ρ = 0.22, *p* = 0.001), DKK-1 levels were positively correlated with Gal-3 levels (ρ = 0.18, *p* = 0.009), and CST levels were positively correlated with fet-A concentration (ρ = 0.20, *p* = 0.004).

### 2.2. Associations of Biomarkers with RF and ACPA Positivity

**Figure 2:**
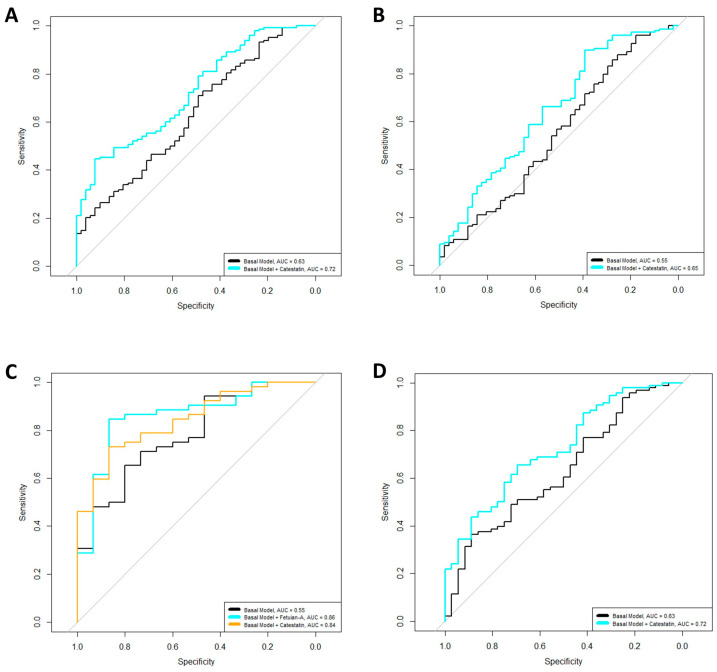
ROC curves of the different logistic regressions. (**A**) Model for rheumatoid factor positivity in the overall cohort; (**B**) model for ACPA positivity in the overall cohort; (**C**) model for rheumatoid factor positivity in male patients; (**D**) model for rheumatoid factor positivity in female patients. The basal models were adjusted for age, sex, BMI, disease duration, use of csDMARDs, NSAIDs, biological drugs, or glucocorticoids, and DAS28-ESR scores.

Multivariate logistic regression models, adjusted for age, sex, BMI, disease duration, DAS28-ESR, and RA treatments, were created to evaluate the associations between the levels of the analysed biomarkers and the presence of ACPAs and RF. We observed that increased levels of CST were associated with increased odds of RF and ACPA positivity (OR = 2.45, *p* = 0.0001 and OR = 1.48, *p* = 0.04, respectively) in the overall cohort. Adding this biomarker to the basal models increased the variability, explained by 8.1% for the RF model and 2.1% for the ACPA model, and improved the model’s qualities by reducing the AICs of the models. Finally, the AUCs of the models also increased when the biomarkers were included (Figure 2A,B). Complete summaries of the models are shown in Table 2.

When the models were stratified by sex, increased levels of CST (OR = 3.20, *p* = 0.03, ∆R^2^ = 9.83) were linked to increased odds of RF positivity in male patients. Conversely, we found that elevated levels of fet-A (OR = 0.38, *p* = 0.03, ∆R^2^ = 7.08) were associated with reduced odds of RF positivity in male patients. The inclusion of these biomarkers in the models not only increased the explained variability but also reduced the AICs of the models and improved the AUCs (Figure 2C,D), thus enhancing overall model quality (Table 2). For female patients, we observed that higher levels of CST were associated with increased odds of RF positivity (OR = 2.40, *p* = 0.001, ∆R^2^ = 7.95). The incorporation of this biomarker into the basal model also led to improved variability explainability, reduced AICs, and enhanced AUCs (Figure 2C,D) (Table 2).

### 2.3. Associations of Biomarkers and Inflammatory Markers of RA

To assess the relationships between the analysed biomarkers and the inflammatory markers of RA (ESR, DAS28-ESR, CRP level, and fibrinogen concentration), we conducted multivariate linear regressions adjusted for age, sex, BMI, disease duration, and RA treatments. In this analysis, we observed a negative association between fet-A levels and the ESR in the overall cohort (β = −0.15, *p* = 0.037). The inclusion of fet-A in the baseline model resulted in an increase in the explained variability (∆R^2^ = 2.03) and a reduction in the AIC, thereby enhancing the overall quality of the model (Table 3).

When we conducted sex-stratified analyses, we observed that fet-A levels were associated with DAS28-ESR in women (β = −0.29, *p* = 0.01, ∆R^2^ = 4.71). Furthermore, in female patients, fet-A levels showed a negative association with ESR (β = −0.25, *p* = 0.004, ∆R^2^ = 5.79) and fibrinogen concentration (β = −0.22, *p* = 0.01, ∆R^2^ = 4.52). The inclusion of these parameters in the models resulted in increased explained variability and reduced AICs, thereby enhancing the overall model quality. Detailed summaries of the models can be found in Table 3.

Finally, when models adjusted for age, sex, BMI, disease duration, and treatments were used to estimate for cPP and cIMT, no associations were found between the new CVD biomarkers and those dependent variables.

## 3. Discussion

In the present work, we studied the relationship of five novel CVD biomarkers (fet-A, DKK-1, Gal-3, IL-32, and CST) with both inflammatory and serological markers of RA and surrogate markers of CVD in a cohort of RA patients. Our multivariate analysis revealed a significant association between CST levels and RF seropositivity across the entire cohort and in men and women separately, as well as between CST levels and ACPA seropositivity in the overall cohort. Adding CST to our models enhanced the AUCs, thereby improving their predictive accuracy. Additionally, RF-positive patients displayed significantly greater CST levels than RF-negative individuals, a trend consistent across both sexes. These findings suggest that CST concentration may be important for improving the prognosis of patients with RA. To our knowledge, the only data on CST levels in RA patients were obtained by Simac et al. in 2022 [47]. Their study revealed significantly elevated CST levels in RA patients compared to controls but did not explore associations between CST levels and RF or ACPA seropositivity. However, their study established significant associations between CST levels and both RA duration and DAS28-ESR scores. These associations were not observed in our study, likely because patients in the Simac cohort were treated with targeted therapies (either bDMARDs or tsDMARDs), whereas only 21.6% of our cohort received treatment with bDMARDs, and because of differences in the model adjustments. Our analysis included additional adjustments for potential confounders, encompassing both treatments and comorbidities, with the objective of eliminating heterogeneity and ensuring that our findings were robust and unaffected by these factors. This adjustment approach strengthens the reliability of our results and supports their applicability across different RA populations.

Recent evidence shows that CST levels may play a significant role in CVD. Research has consistently reported an elevation in CST levels following acute myocardial infarction, which is associated with poorer outcomes [48,49,50]. This increase in expression is thought to act as a compensatory mechanism to counteract the release of catecholamines during CV events, thereby mitigating their adverse effects. In patients with RA who often exhibit autonomic nervous system alterations [51], particularly in those who are double-seropositive for RF and ACPAs and thus have a more severe disease course and prognosis [7,52,53], the increase in CST levels may similarly be an adaptive response to these systemic challenges. We also found that male patients with cPP exhibited elevated CST levels. However, no significant associations were detected between CST levels and surrogate markers of CVD. To our knowledge, no data are available linking CST levels with measurements of CVD surrogate markers in RA patients. Given that the relationships of CST levels with RF and ACPA seropositivity and their potential association with CVD risk remains largely unexplored, further investigation is warranted. This is especially relevant since seropositivity for RF and ACPAs, even in the absence of arthritis, has been linked to CV events [54,55].

Furthermore, our multivariate linear regression analysis showed that fet-A concentration was significantly inversely associated with the ESR in the overall RA cohort. This relationship persisted in women, who additionally exhibited inverse associations of fet-A concentration with fibrinogen levels and DAS28-ESR scores. Additionally, fet-A concentration was linked to lower odds of RF positivity and exhibited a negative correlation with CRP levels and the ESR across the entire RA cohort and with DAS28-ESR scores, fibrinogen levels, and the ESR specifically in women. Overall, these findings suggest that fet-A concentration could be a protective factor against inflammation in individuals with RA, particularly in female patients, and could influence disease activity and severity. Our findings align with those of Sato et al., who reported an inverse correlation between fet-A levels and those of inflammatory markers in RA patients [56]. This relationship is supported by studies showing that IL-6 negatively regulates gene expression and the synthesis of fet-A; specifically, a 75% decrease in IL-6-induced fet-A levels was associated with a 60% increase in CRP levels in the same samples [57]. This is important in RA patients because IL-6 is a key interleukin in this disease [58]. Furthermore, several studies have reported lower fet-A levels in RA patients than in controls [56,59], although the evidence remains controversial; other reports have indicated higher levels of fet-A in RA patients [60,61,62]. However, in patients with CVD, fet-A concentration has been widely studied. Consistent with our results, an inverse association between fet-A concentration and acute-phase reactants has been observed in patients with CVD; interestingly, low concentrations of fet-A and elevated CRP levels were linked to an increased risk of CV-related mortality [24]. Additionally, a large meta-analysis by Xie et al. revealed that high serum levels of fet-A were associated with reduced all-cause mortality in patients with coronary heart disease [23].

Interestingly, while we observed a positive correlation between fet-A concentrations and LDLc levels, a finding consistent with prior studies [63,64], we did not detect an association between fet-A levels and surrogate markers of CVD. To our knowledge, the only available study on the utility of fet-A concentration as a CVD risk biomarker in RA patients showed no association between aortic calcification and fet-A levels in RA patients [56]. Similarly, while no association of fet-A levels with cIMT was observed in the general population, an inverse correlation with coronary artery calcification has also been documented [65]. This suggests that while fet-A concentrations may influence or reflect changes in lipid levels, they do not directly correlate with the common surrogate markers used in this study, which may indicate that the impact of fet-A levels on CVD risk might be more complex or indirect than simply working through changes in lipid levels. Our data highlight the need for further prospective studies evaluating long-term CV outcomes associated with fet-A levels and mechanistic research to understand the fet-A-related pathways involved in these effects.

Regarding Gal-3 and DKK-1, we showed that patients with higher disease activity exhibited elevated levels of Gal-3 and DKK-1, consistent with other studies that have shown correlations between these biomarkers and acute-phase reactants in RA patients [66,67]. However, the literature on Gal-3 levels in RA patients and their modulation by treatments remains controversial [68]. Additionally, our findings revealed that male patients with cPP had lower levels of Gal-3 and DKK-1. In contrast, some studies have linked increased DKK-1 levels with myocardial perfusion defects [44] and Gal-3 levels with subclinical arteriosclerosis in patients with RA [45].

Nonetheless, caution is advised in interpreting these results, as they were not substantiated by multivariate analysis, which showed no significant associations between these biomarkers and inflammatory markers, RF or ACPA seropositivity, or surrogate markers of CVD. We acknowledge the differences between our univariate and multivariate analysis results. However, we emphasise the need for careful interpretation of our univariate findings, as it is possible that the multivariate analysis, by controlling for confounders and considering the combined effects of multiple variables, reveals a more complex underlying relationship than what is observed in the univariate analysis. Additionally, univariate correlations can sometimes appear spurious due to the influence of outliers or values near the detection limit, which can distort the observed relationships, resulting in associations that may not hold up when multiple variables are controlled for in multivariate analysis. This complexity suggests that the associations observed in univariate analysis may not be as robust as those identified through multivariate analysis when considering the broader context of other variables, potentially leading to different conclusions.

In the present study, we observed differences in the analyses stratified by sex, underscoring the physiological variations between men and women and enhancing our understanding of the distinct underlying disease processes in each sex. The exact mechanisms driving these differences remain unclear, but they likely involve a complex interplay of hormonal, genetic, epigenetic, and environmental factors, as well as distinct autoimmune responses, psychosocial stressors, medication effects, body compositions, and comorbidities. These findings highlight the critical need to consider sex as a key variable in RA research. Future studies should aim to integrate a sex-specific perspective to better understand these interactions and develop tailored strategies to mitigate CVD risk in RA patients.

Our study has several limitations. Its cross-sectional design limits our ability to establish causality in the observed associations. While our research focuses on evaluating CV biomarkers within a cohort of patients with RA, the lack of a control group prevents us from directly comparing these findings to a healthy population or other relevant groups. This limitation means that our results should be interpreted with caution, as they do not account for baseline differences that could exist between patients with RA and those without the condition. Additionally, the selection of RA patients from a specific region (European Caucasian) may limit the generalisability of our findings to other populations. Further validation and longitudinal studies are needed to confirm the clinical relevance of the biomarkers we studied. Despite these limitations, the strengths of our statistical analyses suggest that biomarkers, particularly CST and fet-A concentrations, could play significant roles in the prognosis of RA.

In conclusion, our study demonstrates a relationship between various novel CVD biomarkers and RA parameters. Our findings suggest that CST concentration may play an important role in RA prognosis as it is associated with RF and ACPA positivity, while fet-A levels appear to be relevant for assessing inflammation and disease activity. However, these biomarkers are not suitable for evaluating CVD risk through traditional surrogate markers. Although further research is needed, our results suggest that these biomarkers could support the use of autoantibodies and acute-phase reactants in the diagnosis, prognosis, and follow-up of RA patients, and potentially identify patients with lower CVD risk. Continued investigation is essential for clarifying the connection between these biomarkers and CVD risk in RA patients. 

## 4. Methods

### 4.1. Patients

The RA cohort involved in this cross-sectional study has been described in previous works [69,70]. Participants were randomly selected from patients attending the University Hospital Sant Joan de Reus through external consultations, aged 18 to 80 years. Patients with a confirmed RA diagnosis based on medical history, clinical examination, laboratory tests, and imaging, and meeting the American College of Rheumatology (ACR) classification criteria, were included in this study by our rheumatology team. Patients were primarily selected according to the 1987 ACR criteria, with additional consideration of the 2010 criteria to enhance the specificity of patient selection. Patients who initially met the 2010 ACR criteria but later developed other rheumatic diseases were excluded. Exclusion criteria included patients under 18 or over 80 years old, those with acute intercurrent illnesses, or those whose diagnosis had changed. Recruitment was conducted between November 2011 and January 2015, and blood samples were collected on the day of their medical visit. Blood collection and carotid ultrasound were performed on the same day as each patient’s medical visit. As a measure of disease activity, the four-variant Disease Activity Score (DAS28-ESR), used as the local standard of care in our region, was calculated by a team of two highly specialised rheumatologists based on the patient’s erythrocyte sedimentation rate (ESR), the number of tender and swollen joints, and the patient’s global evaluation. To compare highly versus lowly inflamed patients, the DAS28-ESR variable categorised patients with a high inflammatory state (DAS28-ESR ≥ 3.2, including those with moderate and high disease activity levels) and those with a low inflammatory state (DAS28-ESR < 3.2, including patients with low disease activity and those in remission). Pain was measured using a 0–10 visual analogue scale, and patients reported any disability using the health assessment questionnaire (HAQ) index. This study was approved by the Clinical Research Ethics Committee of our hospital (reference: 11-04-28/4proj5), and informed consent was obtained from all patients. The investigation was conducted in accordance with our institution’s guidelines and the principles of the Helsinki Declaration. Patients and the public were not involved in the study’s development.

### 4.2. Clinical Evaluation

Data on the presence of key cardiovascular risk factors, including smoking, BMI, hypertension, diabetes, and dyslipidaemia, along with patient history of cardiovascular events and medication use, were collected. Clinical assessments also included body weight, height, waist circumference, body mass index (BMI), and both systolic (SBP) and diastolic blood pressure (DBP).

### 4.3. Laboratory Measurements

Blood samples from individuals with RA who had fasted for 12 h or more were collected on the same day of each patient’s medical visit. Plasma was obtained by centrifugation of whole blood at 3000 rpm for 10 min, and plasma samples were stored at −80 °C until measurements were performed. The following analytical variables were measured: hemogram, general biochemistry, glycosylated hemoglobin, thyrotropin, albumin, and lipid profile [triglycerides (TGs), total cholesterol (TC), low-density lipoprotein cholesterol (LDLc), high-density lipoprotein cholesterol (HDLc), and very-low-density lipoprotein cholesterol (VLDLc)], using enzymatic methods. Additionally, RF, ACPAs, antinuclear antibodies, and inflammatory markers [ESR, C-reactive protein (CRP), and fibrinogen] were determined using standard methods. RF positivity was defined as an RF value > 20 U/mL (Rheumatoid Factors—II—Roche^®^), and ACPA positivity was defined as an ACPA value > 3 U/mL. Dyslipidaemia (DLP) was defined as TG level > 150 mg/dL and HDLc level < 40 or 50 mg/dL for men and women or treatment with statins or other hypocholesterolaemic drugs.

### 4.4. Ultrasound Evaluation of Carotid Intima-Media Thickness (cIMT) and Carotid Plaque Presence (cPP)

Carotid intima-media thickness (cIMT) was measured using a MyLab 60 X-Vision sonographer (Esaote SpA, Genova, Italy) equipped with a linear array ultrasound probe and a small parts broadband transducer (5–12 MHz). Measurements were taken from the far wall of the common carotid artery (1 cm proximal to the bifurcation) and the internal carotid artery (1 cm distal to the bifurcation) on both the left and right sides. In vivo cIMT measurements were obtained at predefined points using QIMT© radiofrequency image processing software (V 9.03, Esaote SpA, Genova, Italy). To minimise observer variability, all images were acquired and analysed by a single operator. The mean cIMT was determined by averaging three static images from both the left and right carotid arteries. Carotid plaque presence (cPP) was defined as a focal structure encroaching into the arterial lumen by at least 0.5 mm, by 50% of the surrounding IMT value, or with a thickness greater than 1.5 mm.

### 4.5. Biomarker Quantification

ELISA kits were used to quantify biomarkers, adhering to the instructions provided by the manufacturers (Raybiotech, Peachtree Corners, GA, USA).

### 4.6. Statistical Analysis

Continuous variables with a normal distribution are presented as means with standard deviations (SDs), while non-normally distributed continuous variables are presented as medians with interquartile ranges (IQRs). Categorical variables are expressed as percentages and counts. To assess differences between groups, *t* tests, Mann–Whitney U tests, and chi-squared tests were used for normally distributed, non-normally distributed, and categorical variables, respectively. Analyses were performed in the overall cohort and stratified by sex. Pearson and Spearman correlation tests were performed to evaluate the correlations between groups of normally distributed and non-normally distributed continuous variables, respectively. The associations between DAS28-ESR, ESR, and fibrinogen levels (continuous dependent variables) and the studied biomarkers were evaluated with multivariate linear models. For RF positivity and ACPA positivity, multivariate logistic models were applied to estimate their associations with the biomarkers. Receiver operating characteristic (ROC) curves and area under the ROC curve (AUC) values were calculated to evaluate the classification accuracy of each model. All models were adjusted for traditional and known confounders [70], including age, sex, BMI, disease duration, and DAS28-ESR treatment. R^2^, ΔR^2^, and the Akaike information criterion (AIC) were computed for each model. The R^2^ statistic estimates the amount of variability explained by the model, ∆R^2^ shows the increase in variability explained when the different studied biomarkers were included in the models, and the AIC provides the quality of the model, for which a lower AIC value implies better quality. Statistical analyses were conducted using R Studio, version 4.0.1. *p* values less than 0.05 were considered statistically significant.

## Figures and Tables

**Table 1:** Description of the general characteristics, disease features, and treatments of the overall RA cohort, stratified by sex. Normally distributed data are expressed as means and standard deviations (SDs). Non-normally distributed data are expressed as medians and interquartile ranges (IQRs). Categorical data are expressed as percentages. To evaluate differences between groups, a *t* test was used to compare normally distributed data, the Mann‒Whitney U test was used to compare non-normally distributed data, and the chi-squared (*X*^2^) test was used to compare categorical data. *p* values < 0.05 were considered to indicate statistical significance.

	RA (n = 199)	Female (n = 132)	Male (n = 67)	*p*
**Characteristics of the groups**				
Sex—female (%, n)	66%, 132			
Age (years, SD)	57.8 (12.4)	57.3 (12.53)	58.65 (12.17)	0.47
Body mass index (kg/m^2^, IQR)	26.8 (23.2–31.2)	26.5 (22.7–31.6)	27.85 (25.7–30.1)	0.13
Waist circumference (cm, SD)	91.88 (15.11)	88.04 (15.11)	99.45 (12.17)	<0.001
SBP (mmHg, IQR)	135 (120–150)	133.5 (120–148.5)	139 (128–156.5)	0.04
DBP (mmHg, IQR)	80 (71.50–89)	80 (70.75–88)	85 (75–90)	0.02
LDL cholesterol (mg/dL, IQR)	115 (99–135)	115 (97–135.5)	118 (100–134.5)	0.64
HDL cholesterol (mg/dL, IQR)	66 (53.50–75)	69 (61–80.25)	54 (43–66)	<0.001
Triglycerides (mg/dL, IQR)	92 (69–127.5)	88.5 (65.75–125.25)	94 (75–131)	0.31
Glucose (mg/dL, IQR)	89 (82–99)	88 (81–97)	93 (84–102)	0.07
Current smoker (%, n)	27%, 54	28%, 37	25.37%, 17	0.82
Hypertension (%, n)	59.29%, 118	53.03%, 70	71.64%, 48	0.01
Diabetes mellitus (%, n)	11.55%, 23	10.6%, 14	13.43%, 9	0.72
Dyslipidaemia (%, n)	40.70%, 81	39.39%, 52	43.28%, 29	0.71
**Disease features**				
Disease duration (years, IQR)	8 (3–13)	8.5 (3–13.25)	6 (2–11.50)	0.33
DAS28-ESR (median, IQR)	3.43 (2.6–4.26)	3.59 (2.77–4.62)	3 (2.41–3.70)	<0.001
DAS28-ESR ≥ 3.2 (%, n)	56%, 112	65%, 86	39%, 26	<0.001
DAS28-ESR < 3.2 (%, n)	44%, 87	35%, 46	61%, 41	<0.001
HAQ (median, IQR)	0.25 (0–0.75)	0.5 (0.125–0.875)	0 (0–0.25)	<0.001
Rheumatoid factor + (%, n)	74.37%, 148	72.72%, 96	77.61%, 52	0.57
ACPAs + (%, n)	73.86%, 147	74.24%, 98	73.13%, 49	1
ESR (mm/h, IQR)	31 (18.50–50.50)	31 (18.75–54)	29 (18.50–46.50)	0.30
CRP (mg/dL, IQR)	0.5 (0.2–0.9)	0.4 (0.2–0.9)	0.4 (0.2–0.95)	0.68
Fibrinogen (mg/dL, SD)	445.64 (96.53)	442.21 (95.50)	452.40 (98.91)	0.49
cIMT (mm, IQR)	636 (571.8–709.8)	610.2 (565.5–694.2)	667.5 (608.5–744.5)	0.003
**Treatments (%, n)**				
**RA**				
csDMARDs	74.87%, 149	71.21%, 94	82.09%, 55	0.13
Biological agent	21.6%, 43	24.24%, 32	16.41%, 11	0.27
NSAIDs	57.28%, 114	58.33%, 77	55.22%, 37	0.79
Glucocorticoids (Mean dose: 2.91 mg)	51%, 102	52.27%, 69	49.25%, 33	0.80
**Hypertension**				
RAAS inhibitors	24.62%, 49	24.24%, 32	25.37%, 17	0.98
Other	26.13%, 52	22.72%, 30	32.83%, 22	0.17
**Lipid-lowering**				
Statins	16.08%, 32	15.15%, 20	17.91%, 12	0.77
Other	0.15%, 3%	0.15%, 2	0.14%, 1	1
**Growth factors**				
Fetuin A (µg/mL)	273.2 (204.4–354)	289.68 (215.2–355.3)	249 (197.6–342.6)	0.15
DKK1 (ng/mL)	7.30 (5.90–8.56)	7.40 (5.90–8.54)	7.05 (5.89–8.59)	0.77
Galectin-3 (ng/mL)	0.85 (0.02–2.63)	0.92 (0.02–3.26)	0.27 (0.02–2.32)	0.10
IL-32 (pg/mL)	281.76 (412.19)	256.78 (341.41)	330.99 (526.96)	0.30
Catestatin (ng/mL)	7.57 (5.48–9.93)	7.67 (5.75–10.08)	7.04 (5.10–9.40)	0.32

n = number of individuals, SBP = systolic blood pressure, DBP = diastolic blood pressure, LDL = low-density lipoprotein, HDL = high-density lipoprotein, HAQ = health assessment questionnaire index, ACPAs = citrullinated anti-cyclic peptide antibodies, ESR = erythrocyte sedimentation rate, CRP = C-reactive protein, cIMT = carotid intima-media thickness, DAS28-ESR = disease activity score based on ESR, DMARDs = disease-modifying antirheumatic drugs, NSAIDs = nonsteroidal anti-inflammatory drugs, *p* = *p* value.

**Table 2:** Summaries of the multivariate logistic regression models used to estimate the associations between the studied biomarkers and positivity for RF and ACPAs. The basal models were adjusted for age, sex, BMI, disease duration, use of csDMARDs, NSAIDs, biological drugs, or glucocorticoids, and DAS28-ESR scores.

	OR	*p*	R^2^ (%)	AUC	AIC
**Overall cohort**					
**RF positivity**					
Basal Model		0.0001	5.41	0.63	234.25
+**Catestatin**	2.45	0.0001	13.51	0.72	217.92
**ACPA positivity**					
Basal Model		0.01	3.68	0.55	240.18
+**Catestatin**	1.48	0.04	5.71	0.65	237.57
**Male patients**					
**RF positivity**					
Basal Model		0.01	20.32	0.78	74.77
+**Fetuin-A**	0.38	0.03	27.40	0.86	71.73
Basal Model					
**+Catestatin**	3.20	0.03	30.16	0.84	69.76
**Female patients**					
**RF positivity**					
Basal Model		0.001	5.24	0.63	164.58
+**Catestatin**	2.40	0.001	13.19	0.72	154.28

OR = odds ratio, *p* = *p* value, AUC = area under the curve, AIC = Akaike information criterion.

**Table 3:** Summaries of the multivariate linear models used to estimate the associations between the studied biomarkers and inflammatory markers. The basal models were adjusted for age, sex, BMI, disease duration, use of csDMARDs, NSAIDs, biological drugs, or glucocorticoids, and DAS28-ESR scores.

	β	*p*	R^2^ (%)	AIC
**Overall cohort**				
**ESR**				
Basal Model		0.001	10.57	561.50
+**Fetuin-A**	−0.15	0.037	12.60	558.94
**Female patients**				
**ESR**				
Basal Model		0.0001	9.94	377.77
+**Fetuin-A**	−0.25	0.004	15.74	370.99
**DAS28-ESR**				
Basal Model		0.001	9.32	378.68
+**Fetuin-A**	−0.29	0.01	14.03	373.65
**Fibrinogen**				
Basal Model		0.001	5.32	384.38
**+Fetuin-A**	−0.22	0.01	9.82	379.94

*p* = *p* value, AIC = Akaike information criterion.

## Data Availability

The data presented in this study are available on request from the corresponding author due to privacy policies.

## Supplementary Materials

The following supporting information can be downloaded at https://www.mdpi.com/article/10.3390/ijms25189910/s1.
