# Supplementary material for: Enhanced Association of Novel Cardiovascular Biomarkers Fetuin-A and Catestatin with Serological and Inflammatory Markers in Rheumatoid Arthritis Patients
Source: Int J Mol Sci. 2024 Sep 13;25(18):9910. doi: 10.3390/ijms25189910 (PMC11431854; doi:10.3390/ijms25189910)
Supplement: Supplementary file 1 [file ijms-25-09910-s001.zip › ijms-3143757-supplementary.pdf]

Spearman correlations between age and the selected biomarkers		
	**ρ (correlation coefficient with age)**	**p-value**
**Catestatin (ng/mL)**	0.081	0.26
**Galectin-3 (ng/mL)**	-0.0033	0.96
**DKK1 (ng/mL)**	-0.098	0.17
**Fetuin-A (µg/mL)**	-0.14	0.048
**IL-32 (pg/mL)**	-0.075	0.29

Supplementary Table S1. Spearman correlations evaluating the relationship between age and each biomarker concentration in the overall cohort.

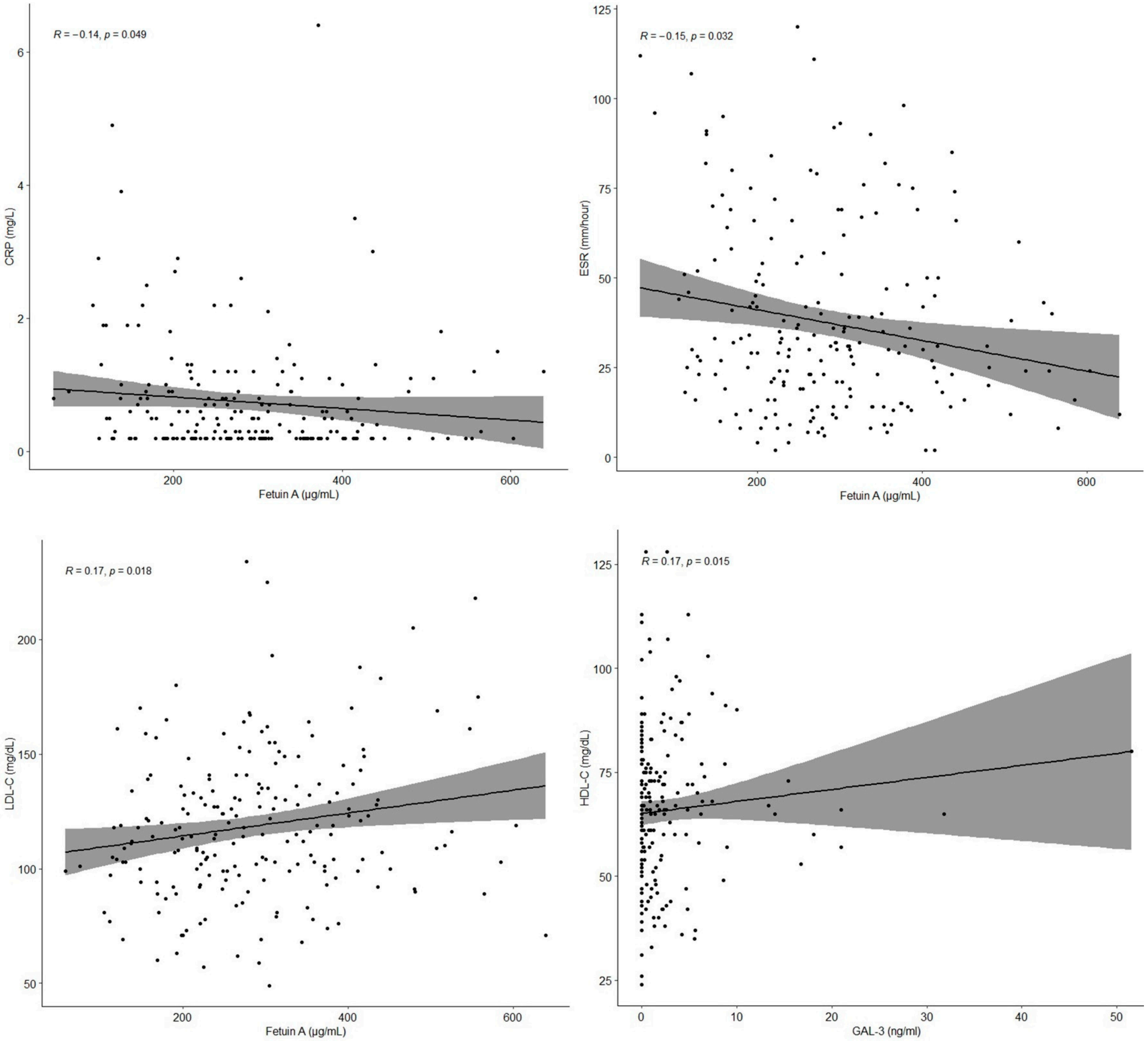

Supplementary Figure S1. Spearman correlations between Fetuin-A and CRP, ESR and LDL-C, and galectic-3 and HDL-C in the overall cohort.

CRP = C-reactive protein, ESR = erythrocyte sedimentation rate, LDL-C = low density lipoprotein cholesterol, HDL-C = high density lipoprotein cholesterol

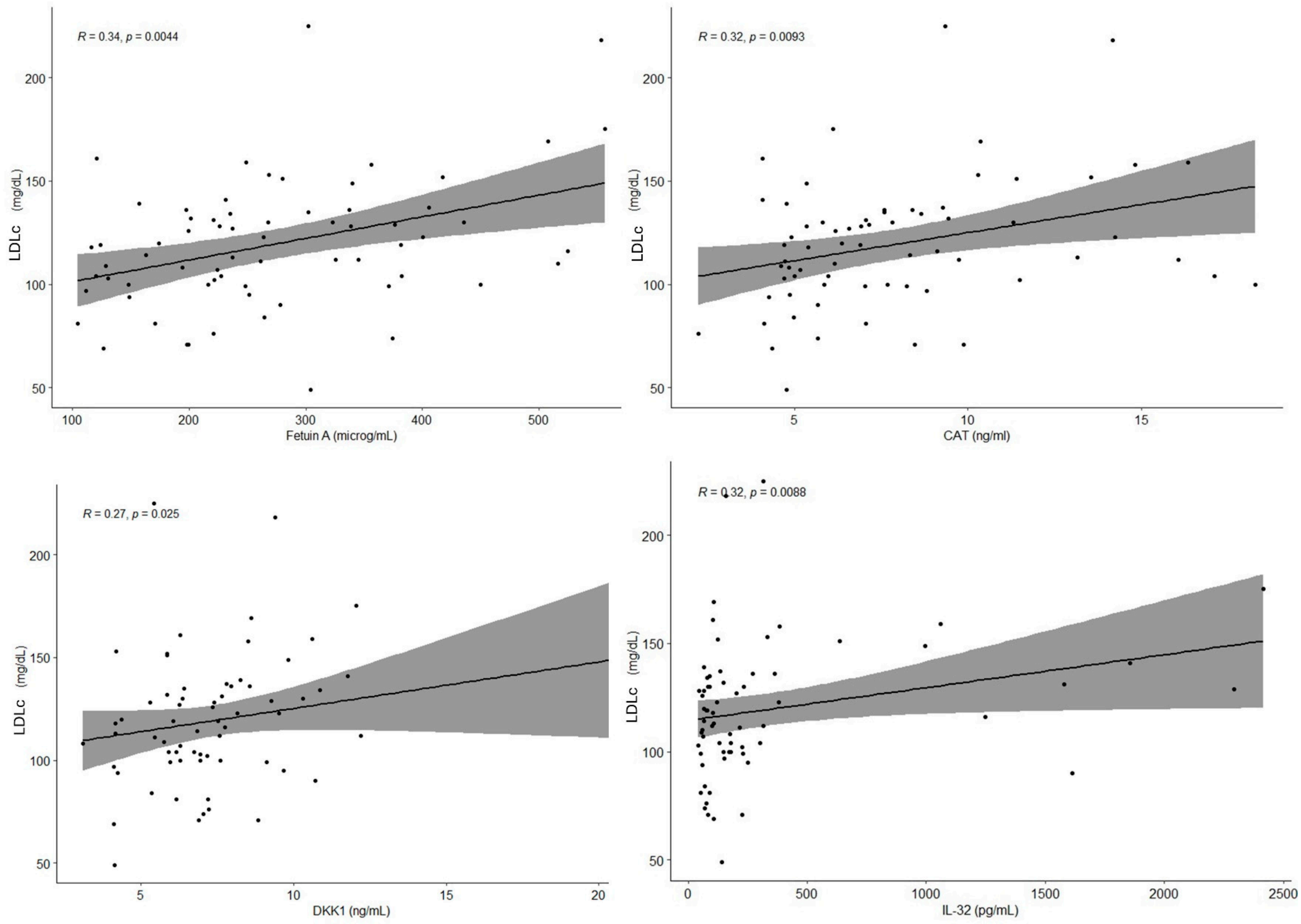

Supplementary Figure S2. Spearman correlations between LDL-C and fetuin A, catestatin, DKK1 and IL-32 in men.

LDL-C = low density lipoprotein cholesterol

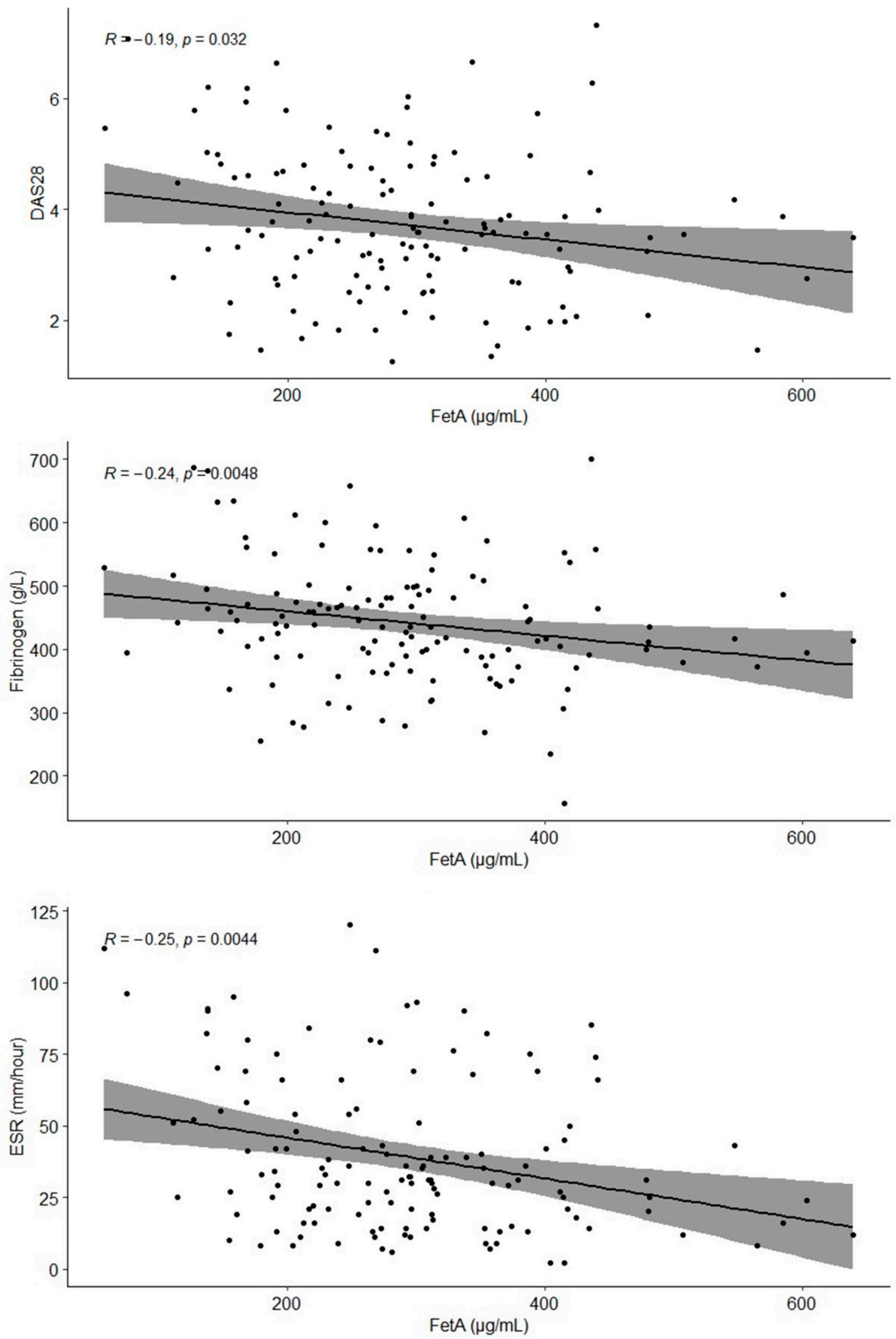

Supplementary Figure S3. Spearman correlations between Fetuin A and CRP, fibrinogen, and DAS28 in women.

CRP = C-reactive protein, DAS28 = disease activity score
